# Carotid Artery Imaging: Insights Into Inflammation and Cardiovascular Disease Risk in Patients With HIV Infection

**DOI:** 10.1161/JAHA.112.001396

**Published:** 2012-04-24

**Authors:** James H. Stein

**Affiliations:** University of Wisconsin School of Medicine and Public HealthMadison,WI

**Keywords:** atherosclerosis, carotid arteries, carotid intima-media thickness, human immunodeficiency virus

As patients with human immunodeficiency virus (HIV) live longer because of advances in antiretroviral therapy, the importance of identifying and preventing non—AIDS-related causes of death has increased. Whether HIV infection, per se, increases risk of cardiovascular disease (CVD) remains an open question, because patients with HIV tend to have higher prevalence of powerful traditional CVD risk factors such as smoking and dyslipidemia, as well as antiretroviral therapy—related risks. Most of the data suggesting that patients with HIV are at increased CVD risk comes from observational studies with important methodological limitations, including short durations of follow-up, low CVD event rates, incomplete ascertainment of risk factors and events, and a lack of HIV-negative controls. Because patients with HIV tend to be relatively young and at low short-term CVD risk, research on CVD risks in patients with HIV has focused heavily on surrogate markers. One technique, carotid ultrasound to measure intima-media thickness (IMT) and assess plaque presence, has been especially useful for studying CVD risk in individuals with HIV. A meta-analysis of 19 cross-sectional studies found significantly higher carotid IMT in individuals with HIV compared to HIV-negative controls, despite significant heterogeneity in published findings.^1^ Limitations aside, the primary findings of the meta-analysis were confirmed in a subsequent study that compared HIV-infected participants in FRAM (the Study of Fat Redistribution and Metabolic Change in HIV Infection) to HIV-negative individuals in MESA (the Multi-Ethnic Study of Atherosclerosis).^2^ Very few studies have evaluated whether HIV infection increases carotid IMT progression, and those studies were small and had mixed results.

On this background, the findings of Hsue et al are important and provocative.^3^ Their findings regarding carotid wall changes are from the largest longitudinal study reported to date (N=300) that included HIV-negative controls (n=47). Its median length of follow-up (2.4 years) was more than twice as long as a previous report from their group.^4^ Their main findings were (1) HIV-positive participants had higher carotid IMT and more prevalent carotid plaque than those without HIV infection; (2) progression of carotid IMT was more rapid in HIV-infected individuals, especially in the carotid bifurcation (or “bulb”) region; (3) high-sensitivity C-reactive protein (hsCRP), a nonspecific marker of inflammation, was elevated in individuals with HIV; and (4) increased hsCRP was associated with progressive wall thickening in the carotid bifurcation region. Limitations of this study include its small number of HIV-negative controls and important between-group differences in CVD risk factors and carotid IMT at baseline. To the extent that carotid IMT progression depends on baseline IMT and risk factor exposure, differences in IMT progression rates may simply reflect baseline IMT differences. However, their finding of greater rates of incident plaque among HIV-positive individuals is unique and suggests that HIV seropositivity accelerates atherogenesis. This observation is especially notable because it was observed *after* exclusion of individuals with plaque at baseline. The authors’ hypothesis—that individuals with HIV infection are at increased risk for accelerated atherosclerosis due to persistent inflammation—is important and supported by their work.

In order to understand the pathophysiology of arterial injury in patients with HIV, it is important to recognize that in the absence of plaque, carotid IMT can increase because of *intimal and/or medial proliferation*, which can be normal or pathological.^5,6^ “Normal” aging leads to both intimal and medial proliferation.^5,6^ Adaptive intimal thickening is a response to changes in blood flow, wall tension, or lumen diameter that start after birth.^5^ It is characterized pathologically by increases in vascular smooth muscle cells, proteoglycans, and collagen with rare inflammatory cells.^6^ Adaptive intimal thickening is distinguished from pathological intimal thickening—the precursor of atherosclerosis—by the presence of lipid pools.^6^ Because current carotid vascular ultrasound techniques lack the resolution to distinguish between adaptive and pathological wall thickening, increased carotid IMT should be described more precisely as “arterial injury” rather than “atherosclerosis.”^5,7^ However, when a focal area of carotid wall thickness exceeds 1.5 mm, atherosclerotic plaque generally is present.^7^

The terminology here is important, as pathology studies have demonstrated differential prevalence of atherosclerosis by carotid segment, and observational studies have demonstrated segmental differences in carotid IMT and IMT progression rates. Atherosclerosis tends to develop earliest in the carotid bifurcation and proximal internal carotid artery segments, whereas common carotid artery atherosclerosis tends to occur late and is less common. Similarly, carotid IMT is thickest and progresses most rapidly in the bifurcation region but progresses more slowly in the common carotid artery. These observations likely are due to changes in fluid dynamics from a laminar flow pattern in the common carotid artery to more turbulent flow with low wall shear stress in the bifurcation and proximal internal carotid segments. Although some studies have suggested differential CVD risk factor effects on certain carotid segments, no segment-specific risk factor effects have been consistently observed in large population-based studies, so differences between studies likely reflect differences in underlying populations, statistical modeling, or imaging techniques. In the largest report that compared individuals with and without HIV infection (FRAM and MESA), all of the traditional CVD risk factors were associated with increasing internal carotid and common carotid IMT, including HIV infection. Because carotid IMT progression rates are small relative to the effects of risk factors and technical variability, it is not a surprise that smaller studies variably show or do not show risk factor effects in certain segments. Similarly, because all CVD risk factors appear to influence wall thickening in all segments, large observational studies also have demonstrated that the CVD risks associated with increased carotid IMT are similar among the 3 carotid segments.^7–11^ Therefore, the association between hsCRP levels and carotid bifurcation IMT observed by the authors^3^ is less about location than it is about the pathophysiology of arterial injury. If the association between hsCRP and carotid IMT progression is real, the rich carotid IMT literature suggests that the association will be true in all segments, given a large enough sample size and sufficient duration of follow-up and technical rigor.

That inflammation influences atherogenesis is widely accepted; however, it is quite possible that the effects of inflammation on carotid wall injury are enhanced by HIV infection, a disease characterized by persistent inflammation and immune activation. In the Women's Interagency Health Study, markers of T-cell activation and senescence were associated with carotid artery lesions and carotid stiffness.^12,13^ In a post hoc analysis of data from a randomized antiretroviral therapy initiation study, increased levels of hsCRP and interleukin-6 predicted death,^14^ and in an observational study, both HIV seropositivity and hsCRP predicted acute myocardial infarction.^15^ These reports and that of Hsue et al^3^ suggest that the inflammation associated with HIV accelerates arterial injury. Of course, it really is no surprise that arterial wall and serological markers of inflammation are increased in patients with HIV, because atherosclerosis and HIV are inflammatory diseases, and “inflammation” is a very broad term, encompassing a wide range of immunologic and metabolic processes.

Because most of the markers examined in individuals with or at risk for CVD are nonspecific, there is a great deal of research that needs to be conducted to better understand the pathophysiology of arterial injury and CVD risk in individuals with HIV. A major challenge is to determine if the presence of inflammation or immune activation in patients with HIV predicts CVD risk, and if so, which markers are most predictive. Meeting this challenge will require large, prospective, longitudinal studies of CVD risk factors and outcomes in representative cohorts of HIV-infected and HIV-negative individuals. To identify these associations likely will require several thousand subjects, more than a decade of follow-up, reliable biomarker/imaging tests, and strict endpoint adjudication. This challenge also could be addressed by randomized clinical trials of interventions that reduce inflammation or immune activation. Another challenge is determining which inflammatory pathways are most relevant to atherogenesis and if they differ in patients with HIV infection. Addressing this challenge will require both mechanistic studies in animal models of atherosclerosis and translational studies using arterial imaging that can directly assess arterial inflammation and plaque vulnerability. Finally, it is not known, even in patients without HIV infection, if treating inflammation without improving other risk factors reduces CVD risk. Randomized clinical trials of nonspecific anti-inflammatory agents or those that reduce immune activation, immune senescence, or viral reservoirs will eventually be studies that most influence science and patient care. Randomized clinical trials also will be needed to disentangle the effects of antiretroviral therapy on CVD risk from those of underlying CVD risk factors and HIV infection.

We know a great deal about atherosclerosis in the general population, and much of that knowledge is transferrable to patients with HIV. HIV seropositivity probably is a marker of increased CVD risk; however, we still do not know the magnitude of the increased CVD risk having HIV infection imparts, its mediators, or the best interventions to prevent CVD in patients living with HIV.
